# Multi-Enzymatic Cascade One-Pot Biosynthesis of 3′-Sialyllactose Using Engineered *Escherichia coli*

**DOI:** 10.3390/molecules25163567

**Published:** 2020-08-06

**Authors:** Zhongkui Li, Zhijian Ni, Xiangsong Chen, Gang Wang, Jinyong Wu, Jianming Yao

**Affiliations:** 1 Institute of Plasma Physics, Hefei Institutes of Physical Science, Chinese Academy of Sciences, Hefei 230031, China; xx1922@mail.ustc.edu.cn (Z.L.); mars_pharma@163.com (Z.N.); xschen@ipp.ac.cn (X.C.); 2 University of Science and Technology of China, Hefei 230026, China; 3 Huainan New Energy Research Center, Institute of Plasma Physics, Hefei Institutes of Physical Science, Chinese Academy of Sciences, Huainan 232063, China; 4 Wuhan Zhongke Optics Valley Green Biotechnology Co.,Ltd., Wuhan 430200, China; ayurnero@163.com

**Keywords:** multi-enzymes, biosynthesis, 3′-SL, cofactor regeneration

## Abstract

Among the human milk oligosaccharides (HMOs), one of the most abundant oligosaccharides and has great benefits for human health is 3′-sialyllactose (3′-SL). Given its important physiological functions and the lack of cost-effective production processes, we constructed an in vitro multi-enzymatic cofactor recycling system for the biosynthesis of 3′-SL from a low-cost substrate. First, we constructed the biosynthetic pathway and increased the solubility of cytidine monophosphate kinase (CMK) with chaperones. We subsequently identified that β-galactosidase (*lacZ*) affects the yield of 3′-SL, and hence with the *lacZ* gene knocked out, a 3.3-fold increase in the production of 3′-SL was observed. Further, temperature, pH, polyphosphate concentration, and concentration of divalent metal ions for 3′-SL production were optimized. Finally, an efficient biotransformation system was established under the optimized conditions. The maximum production of 3′-SL reached 38.7 mM, and a molar yield of 97.1% from N-acetylneuraminic acid (NeuAc, sialic acid, SA) was obtained. The results demonstrate that the multi-enzymatic cascade biosynthetic pathway with cofactor regeneration holds promise as an industrial strategy for producing 3′-SL.

## 1. Introduction

Human milk oligosaccharides (HMOs) are the third most abundant component in human milk after lactose and fat [1,2] and show important biological functions. Sialyllactose (SL) is an acidic human milk oligosaccharide that can be divided into 3′-SL and 6′-SL according to the position at which sialic acid binds to the lactose moiety [3]. Several beneficial effects of 3′-SL on infants have been demonstrated, including neutralization of toxins produced by enteric bacteria [4,5], preventing bacterial or viral adhesion to the epithelial surface, [6] and prebiotic effects [7,8]. Due to important biological functions and a lack of cost-effective industrial production methods, the production of 3′-SL from low-cost substrates is necessary.

Considering the tedious steps of chemical synthesis, some methods for 3′-SL preparation using enzymatic or whole-cells have been proposed [9,10]. Moreover, 3′-SL can be produced by sialyltransferase and trans-sialidase enzymes. It is known that trans-sialidase transfers α-2, 3-linked sialic acid from casein glycomacropeptide (cGMP) to lactose and forms 3′-SL [10,11,12]. In the previous study, although the substrate catalyzed by trans-sialidase was cheap, the catalytic yield of 3′-SL and substrate utilization were low.

Sialyltransferase transfers SA from an activated nucleotide sugar donor to an acceptor oligosaccharide. The activated nucleotide sugar is expensive and limits the generation of 3′-SL. To overcome this problem, several methods with regeneration systems have been developed. Endo et al. [13] used a coupled microbial system for the biosynthesis of cytidine monophosphate N-acetylneuraminic acid (CMP-NeuAc) and 3′-SL. The production of 3′-SL reached 52 mM with 85% yield from sialic acid after an 11 h reaction. This led to the highest concentration of 3′-SL production. However, by coupling this method with a whole-cell catalytic reaction, the conversion rate was low. Gilbert et al. [14] utilized CMP-NeuAc synthetase and sialyltransferase fusion enzyme to obtain 3′-SL from sialic acid and lactose, which involved cytidine triphosphate (CTP) generation from adenosine triphosphate (ATP), phosphoenolpyruvate (PEP), and cytidine monophosphate (CMP). Moreover, Nah’alka [15] found a novel polyphosphate kinase to regenerate CTP in a two-step biosynthesis of 3′-SL. The relevant research results mentioned above are listed in Supplementary Table S1. Although some research results already have good 3′-SL production, the overall reaction time and substrate economics need to be further improved.

**Figure 1:**
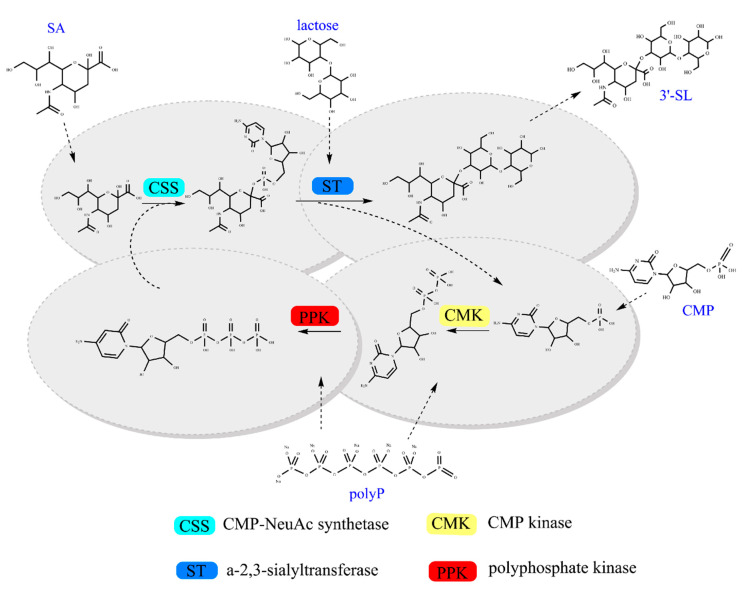
Overview of the multi-enzyme cofactor recycling pathway for 3′-SL production.

In this study, we propose a coupled cell-free multi-enzyme one-pot system with CTP regeneration for the biosynthesis of 3′-SL. To obtain 3′-SL with high efficiency and conversion rate, we optimized the protein expression, reaction conditions, and concentration of cofactors in a genetically modified *E. coli* strain. Further, the optimized conditions were applied to investigate the production of 3′-SL from CMP, lactose, and SA (Figure 1).

## 2. Results and Discussion

### 2.1. Construction of the Biosynthetic Pathway of 3′-SL

CMP-NeuAc synthetase (CSS) converts SA and CTP to CMP-NeuAc. Sialyltransferase (ST) catalyzes the transfer reaction between CMP-NeuAc and lactose to generate 3′-SL. Furthermore, the released CMP is exploited to regenerate CTP by CMP kinase (CMK) and polyphosphate kinase (PPK) which catalyze the reaction.

### 2.2. Protein Expression and Solubility Analysis

**Figure 2:**
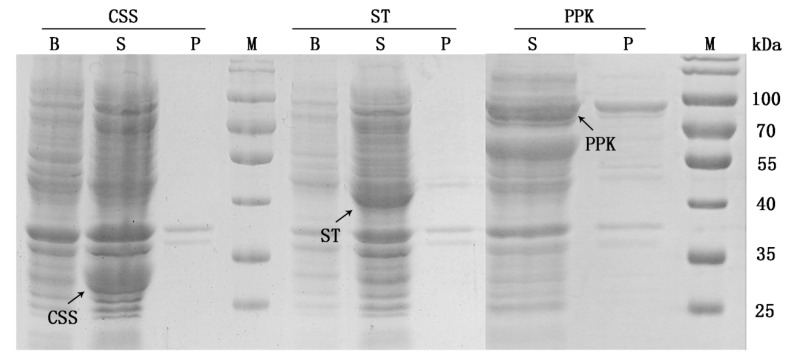
SDS-PAGE of the expressed proteins of CSS, ST, and PPK. Lane S denotes supernatant of the cell-extracts, lane P denotes precipitation fraction of the cell-extracts; lane B denotes cell lysate before induction; lane M shows molecular weight markers.

**Figure 3:**
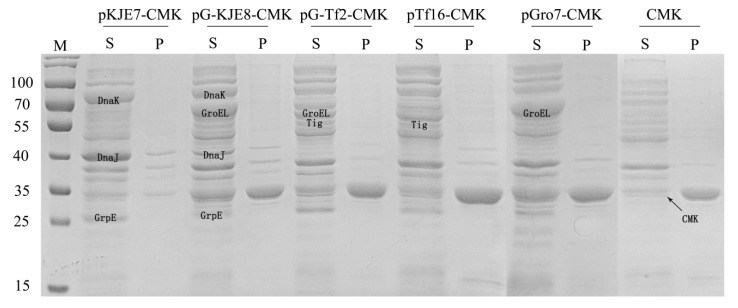
SDS-PAGE of CMK co-expression with various chaperones. Lane S denotes supernatant of the cell-extracts, lane P denotes precipitation fraction of the cell-extracts; lane M shows molecular weight markers.

To enhance the expression of each protein in the synthetic pathway of 3′-SL, target genes were inserted into an expression plasmid under the control of the T7 promoter. Sodium dodecyl sulfate-polyacrylamide gel electrophoresis (SDS-PAGE) was used to determine the expression status of the recombinant proteins. As shown in Figure 2, the strains carrying the plasmids pET-CSS, pET-ST, and pET-PPK expressed soluble proteins. Further, CSS, ST, and PPK had molecular weights of approximately 25, 42, and 80 kDa, respectively, which is consistent with the predicted molecular masses. However, CMK showed low solubility even after optimizing the expression condition, as shown in Figure 3 where less than 10% of the total protein was detected as a soluble protein. A previous study suggests that wild-type CMK does have the low solubility [16], although lowering the temperature at which the induction of the protein expression is performed can promote better solubility of the protein. However, this will result in lower biomass production which increases the burden on the fermentation process. Moreover, the enzyme activities of each enzyme were determined (Supplementary Table S2). The results showed that specific enzyme activities could be detected in the corresponding cell extracts. At the same time, since CMK and PPK were derived from *E. coli*, there was background enzyme activity tested in cell extracts without expression plasmids.

### 2.3. An Increase in CMK Solubility by Co-Expression with Chaperones

**Figure 4:**
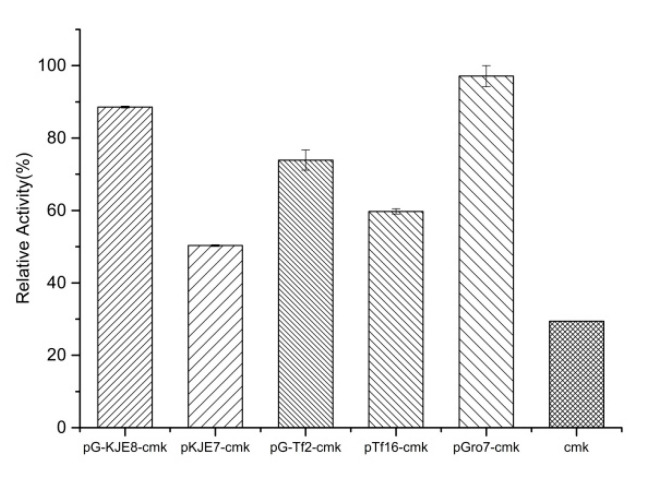
Effect of CMK co-expression with different molecular chaperones.

Protein solubility is the basis for efficient enzyme catalysis or whole-cell catalysis. To increase the soluble expression of CMK, molecular chaperones that are known to increase the solubility of heterologous proteins were employed [17,18]. By comparing the co-expression of five molecular chaperones with the target protein (Figure 4), pGro7 was found to enhance solubility by approximately 50% of the total protein, which demonstrated the highest solubility as compared to other chaperons and the control. Compared with the control, the co-expression of CMK and pGro7 increased the enzyme activity of the target protein by 3.4 times.

### 2.4. Effect of β-Galactosidase Gene Deletion on the Production of 3′-SL

**Figure 5:**
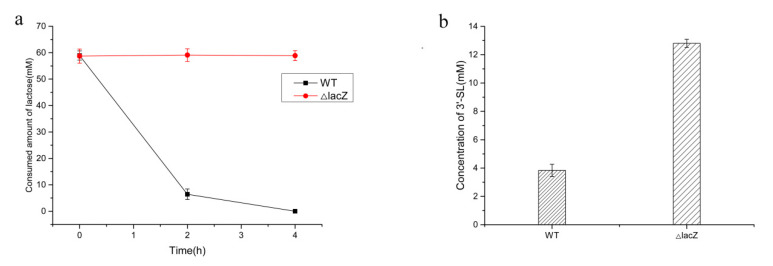
Effect of β-galactosidase deletion. (**a**) Effect of β-galactosidase deletion on the degradation of lactose; (**b**) Effect of β-galactosidase deletion on the production of 3′-SL.

It is known that β-galactosidase is present in wild-type *E. coli*, which can break down lactose into galactose and glucose [19,20]. However, lactose is an important substrate for the conversion of 3′-SL; hence, the degradation of lactose has an adverse effect on the accumulation of 3′-SL. The optimal amount of cell extracts added was investigated before starting to verify the lactose degradation (Supplementary Figure S1). With the amount of cell extracts gradually increased, the 3′-SL content also showed an increasing trend, but there was no significant difference in 3′-SL content at the amount of 40 g/L and 48 g/L added, so the concentration of 40 g/L was used. 60 mM lactose can be completely degraded by wild-type *E. coli* within 4 h (Figure 5a). Under the same lactose concentration, in a genetically manipulated strain with the galactosidase gene knocked out, no obvious lactose content change was observed (Figure 5b). By knocking out the β-galactosidase gene of wild-type *E. coli*, the amount of 3′-SL increased by a 3.3-fold, indicating that elimination of the lactose decomposition pathway was beneficial for the synthesis of 3′-SL.

### 2.5. Effect of pH, Temperature, and Metal Ions on the Production of 3′-SL

**Figure 6:**
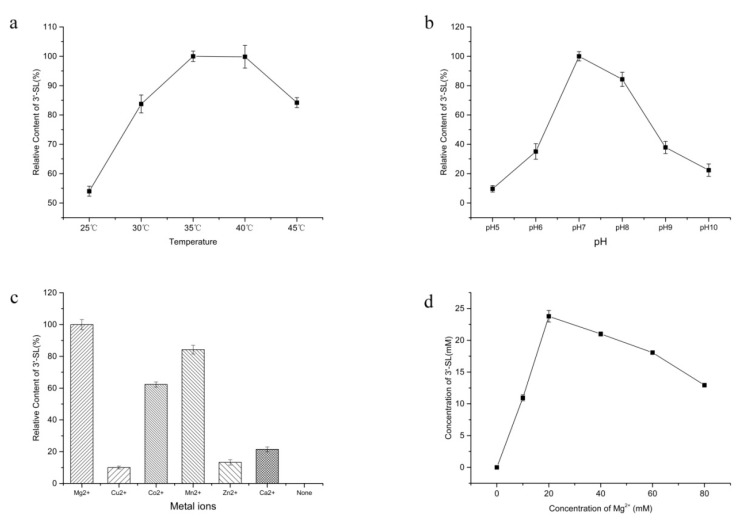
Effect of pH, temperature, and metal ions on the production of 3′-SL. (**a**) Effect of temperature on the reaction rate of 3′-SL biosynthesis; (**b**) Effect of pH on the reaction rate of 3′-SL biosynthesis; (**c**) Effect of different metal ions on the reaction rate of 3′-SL biosynthesis; (**d**) Effect of Mg^2+^ concentration on the production of 3′-SL.

Since each enzyme involved in the biosynthetic pathway has different optimal conditions, they were further studied (Figure 6). During temperature optimization experiments, the other components in the reaction system were fixed to pH 7, 50 mM SA, 60 mM lactose, 20 mM polyphosphate, 20 mM MgCl_2_, 20 mM CMP, and 40 g/L of each cell extracts. As shown in Figure 6a, the relative content of 3′-SL was reduced when the temperature was lower than 35 °C or higher than 40 °C. Further, high relative content was displayed at 35 °C and 40 °C. Considering the fact that energy consumption required the maintenance of high temperatures, 35 °C was selected as a suitable reaction temperature.

The reaction rate at various pH was evaluated, and the other components in the reaction system were fixed to 35 °C, 50 mM SA, 60 mM lactose, 20 mM polyphosphate, 20 mM MgCl_2_, 20 mM CMP, and 40 g/L of each cell extracts. In the present study, the optimal pH of the reaction was found to be 7.0 (Figure 6b), and the production of 3′-SL decreased sharply in conditions lower or higher than pH 7. Previously, an optimal pH with CSS from *Neisseria meningitides* was found to be 8.5 [21] and with ST, the optimal range of pH was found to be 5.8–8.0 [22]. At pH 5, the reaction rate was 10% of the optimal rate and the rate further decreased to 20% at pH 10. This indicates that pH should be controlled within a certain range to maintain the optimal reaction rate.

Divalent metal ions are necessary cofactors for some enzymes to catalyze reactions [23]. Therefore, the effect of different metal ions on enzyme activity was investigated (Figure 6c). The other components in the reaction system were fixed to 35 °C, pH7, 50 mM SA, 60 mM lactose, 20 mM polyphosphate, 20 mM CMP, and 40 g/L of each cell extracts. The enzyme activity when Mg^2+^ was used as a cofactor was defined as 100%. Further, the catalytic activities of the enzyme with Mn^2+^ and Cu^2+^ as cofactors were 85% and 10% of the maximum activity, respectively. Meanwhile, no activity was detected in the absence of any metal ion. The results showed that Mg^2+^ was the most suitable and necessary metal ion.

Furthermore, the effect of Mg^2+^ concentration on 3′-SL production was examined (Figure 6d). The 3′-SL yield decreased sharply when the Mg^2+^ concentration was lower or higher than 20 mM. Hence, 20 mM Mg^2+^ was chosen as the optimal metal ion concentration for subsequent optimization experiments.

### 2.6. Effect of CMP and Polyphosphate Concentrations on the Production of 3′-SL

**Figure 7:**
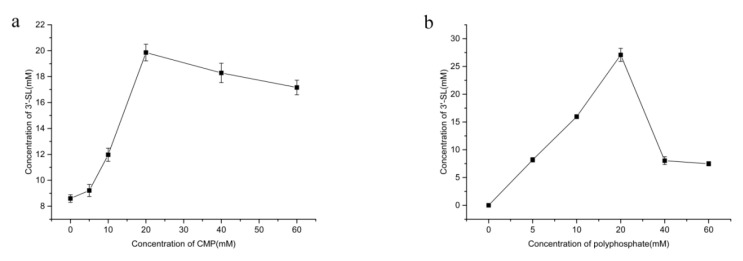
Effect of CMP and polyphosphate concentration on the production of 3′-SL. (**a**) Effect of CMP concentration on the production of 3′-SL; (**b**) Effect of polyphosphate concentration on the production of 3′-SL.

CMP concentration gradients were set from 0–60 mM, other ingredients were regulated to 35 °C, pH = 7, 50 mM SA, 60 mM lactose, 20 mM polyphosphate, 20 mM MgCl_2_, and 40 g/L of each cell extracts. The concentration of 3′-SL increased rapidly with the increase in CMP concentration (Figure 7a) and reached a maximum when 20 mM CMP was added. However, the 3′-SL yield was decreased when the concentration of CMP in the reaction mixture was higher than 20 mM, indicating that CMP inhibited the reaction when the concentration was higher than the optimal concentration. This is consistent with previous studies, which showed a similar inhibitory effect of CMP on the reaction [22,24]. It should be noted that, in the absence of CMP, 8.5 mM of 3′-SL was produced. This could be because CMP was produced by the degradation of the endogenous RNA in the cell-free extracts [25,26], suggesting that RNA could be utilized as the natural raw material for the production of 3′-SL and can thus reduce the production costs.

To evaluate the effect of polyphosphate content on 3′-SL formation, 35 °C, pH7, 50 mM SA, 60 mM lactose, 20 mM CMP, 20 mM MgCl_2_, and 40 g/L of each cell extract were used (Figure 7b). A positive correlation between 3′-SL concentration and polyphosphate concentration were found until the polyphosphate concentration reached 20 mM when the 3-SL production was the highest. Therefore, the optimum concentration of polyphosphate was found to be 20 mM. However, the concentration of 3′-SL decreased sharply when polyphosphate concentration was higher than 20 mM, as it can chelate the metal ions [27]. On the one hand, as the concentration of polyphosphate increased, leading to the loss of essential Mg^2+^ ions and affecting the 3′-SL catalysis. On the other hand, when no polyphosphate was added to the reaction system, no product was detected, indicating that polyphosphate was an essential substrate for catalyzing the reaction [28].

### 2.7. Effect of the Ratio of Cell Extracts on the Production of 3′-SL

**Figure 8:**
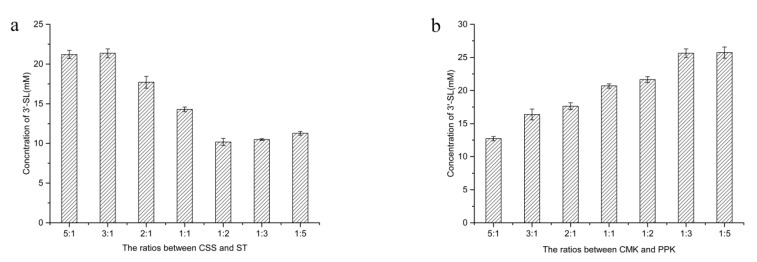
Optimization of the ratios between four cell-free extracts for 3′-SL biotransformation. (**a**) the ratios between CSS and ST; (**b**) the ratios between CMK and PPK.

Due to the expression levels of each enzyme are different, the same amount of cell extracts might have an impact on the production of 3′-SL. The 3′-SL production did not increase with the addition of the ST cell extracts increased, but as the addition of CSS increased to 3 times the initial concentration, the 3′-SL production increases by 49%. However, with the addition of CSS was further increased, the 3′-SL production did not increase significantly (Figure 8a). Hence, the optimal ratios between CSS and ST was set as 3:1, which contained 120 g/L CSS and 40 g/L ST.

Moreover, with the addition of PPK increased to three times the initial concentration, the 3′-SL production increased by 24%, and the production did not further improve as to PPK increased (Figure 8b). Interestingly, when the addition of CMK was increased to five times the initial concentration, the 3′-SL production not only failed to increase, which resulted in a 38% decrease in content. This may be due to the amount of CMK addition increased, which affected the ratio of CMK and PPK in CTP regeneration, and further affected the 3′-SL formation. Therefore, the optimal ratios between CMK and PPK were determined as 1:3, which contained 40 g/L CMK and 120 g/L PPK. Overall, the optimal ratio of four cell-free extracts was obtained. Combined with the above optimal transformation conditions, it is could be used to investigate the production of 3′-SL in cell-extracts.

### 2.8. Production of 3′-SL under Optimal Conditions

**Figure 9:**
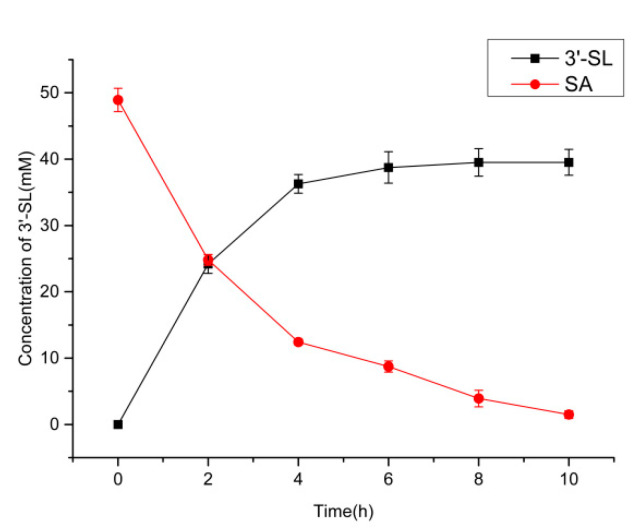
Time course of 3′-SL production under optimal conditions.

Based on the optimized conditions, a 25 mL conversion system was used to investigate the production of 3′-SL (Figure 9). The amount of 3′-SL in the reaction system increased rapidly with a decrease in SA concentrations. After 6 h of the start of the reaction, 38.7 mM of 3′-SL was detected and no more 3′-SL was detected later. To summarize, 38.7 mM of 3′-SL was produced from 48.9 mM SA (the initial actual content of the reaction) in 6 h, with a productivity rate of 6.45 mM/h. After considering the remaining amount of SA, the yield of 3′-SL was found to be 97.1%. Although the production of 3′-SL ceased after 6 h, the SA content gradually decreased. This could be because of the background activity of intact *nan*A gene expression, which is an endogenous gene of *E.coli* and allows the cleavage of SA to pyruvate and N-acetylmannosamine, resulting in a decrease in SA content. This suggests that an additional gene disruption step could lead to better substrate utilization.

In addition, it should be pointed out that no significant product degradation was detected in the reaction mixture, which verified that the α-2, 3-sialyltransferase from *Neisseria gonorrhoeae* had no sialidase activity. In this catalytic reaction, sialyltransferase with mono-function was preferred, which does not produce by-products during the conversion process. Other sialyltransferases with multiple functions should be avoided so as to obtain high-yield products [29].

A few 3′-SL catalytic synthesis methods with an intermediate product generation such as CMP-NeuAc using cofactor regeneration have been proposed [30,31,32]. However, the proposed cofactor regeneration methods made use of either expensive substrates or low catalytic efficiency, which hinder industrial production. Owing to these shortcomings, the use of polyphosphate, CMK, and PPK seems to be a practical method of CTP regeneration.

Although our study has certain advantages over the previously described methods, the 3′-SL output was low, and fermentation with four different cells was cumbersome and time-consuming. It is desirable to decrease the number of cells for the pilot-scale production of 3′-SL. Hence, to overcome this obstacle, we are in the process of constructing multiple enzyme genes into a single cell for biocatalysts. At the same time, to reduce the burden of cell fermentation and conversion process, we are also considering improving the permeability of the cell and immobilizing the whole cell to increase the number of cell usage.

## 3. Materials and Methods

### 3.1. Materials

CMP-NeuAc and 3′-sialyllactose were purchased from Carbosynth (Carbosynth China). SA was kindly provided by CASOV (Wuhan, China) and CMP was purchased from Huaren (Wuhu, China). All other chemicals used in the study were commercially available and were of analytical grade.

### 3.2. Plasmids and Strains

The plasmids and strains used in this study are listed in Table 1. *E. coli* BL21 Star (DE3) and *E. coli* BL21 Star (DE3) Δ*lac*Z were used as the host strain for protein expression. Chromosomal gene disruption of the host strain was carried out with the λ red homology recombination method [33]. The genes used in this study were inserted into the expression plasmid pET-22b (+) between HindIII and BamHI restriction sites with full-length codon-optimization and synthesized by General Biosystems (Chuzhou, China). Molecular chaperones were purchased from Takara (Dalian, China) and transformed into *E. coli* BL21 Star (DE3) competent cells by chemical transformation.

### 3.3. Protein Expression and Optimization

The pET-CSS, pET-ST, pET-CMK, and pET-PPK recombinant plasmids were dissolved in distilled water and separately transformed into *E. coli* BL21 Star (DE3). The recombinant strains were cultured overnight at 37 °C in Luria-Bertani (LB) medium containing 50 μg/mL ampicillin (Amp). Subsequently, 1% volume of the overnight culture was added to a fresh LB medium containing 50 μg/mL Amp. The recombinant proteins were induced when OD_600_ reached 0.6–0.8 by adding isopropyl-thiogalactopyranoside (IPTG) to a final concentration of 0.1 mM, followed by incubation at 20 °C for 12 h.

For protein optimization, the strains were co-expressed with different molecular chaperones and cultured with 50 μg/mL Amp and 30 μg/mL chloramphenicol (Cm) (Table S3). Furthermore, 5 mg/mL L-arabinose was used to induce the expression of the plasmids pGro7, pKJE7, and pTf16. Moreover, tetracycline (Tet) was used to induce the expression of the plasmid pG-Tf2 at a final concentration of 5 ng/mL. The plasmid pG-KJE8 was induced by both L-arabinose and Tet at a final concentration of 5 mg/mL and 5 ng/mL, respectively. When the culture OD_600_ reached 0.6–0.8, the inducer corresponding to each molecular chaperone and 0.1 mM IPTG was added to induce the protein expression by incubating the culture for 12 h at 20 °C.

The cells were collected after centrifugation at 10,000× *g* and 4 °C for 10 min. This was followed by a wash step with 50 mM pH 8.0 Tris-HCl solutions and lysis of the cells by sonication on an ice bath. The lysate was centrifuged to separate the soluble and insoluble fractions. Each fraction was examined by SDS-PAGE.

### 3.4. Enzyme Activity Assays

CSS activity was measured according to the method with a slight modification. The reaction mixture included 0.2 M Tris-HCl (pH 8.5), 20 mM MgCl_2_, 5 mM NeuAc, 5 mM CTP and an enzyme sample. The reactions were performed at 37 °C for 10 min. One unit of enzyme activity was defined as the amount of enzyme that catalyzes the formation of 1 μmol CMP-NeuAc per min.

Sialytransferase activity was measured combine with CMP-NeuAc synthetase, the reaction included 0.2 M Tris-HCl (pH 8.5), 20 mM MgCl_2_, 5 mM SA, 5 mM CTP and 10 mM lactose. Both CMP-NeuAc synthetase and sialyltransferase were added into the system to start the reaction. The reactions were performed at 37 °C for 30 min. One unit of enzyme activity was defined as the amount of enzyme that catalyzes the formation of 1 μmol 3′-SL per min.

The CMP kinase and polyphosphate kinase activity were measured according to a previously described method [28]. The CMP kinase activity reaction mixture included 50 mM Tris-HCl (pH 8.0), 50 mM (NH_4_)_2_SO_4_, 10 mM MgCl_2_, 10 mM ATP, and 5 mM CMP. The polyphosphate kinase activity reaction mixture included 50 mM Tris-HCl (pH8.0), 50 mM (NH_4_)_2_SO_4_, 10 mM MgCl_2_, 5 mM ADP and 10 mM hexametaphosphate. The reactions were performed at 30 °C for 30 min. One unit of enzyme activity was defined as the amount that catalyzes the formation of 1 μmol cytidine diphosphate (CDP)/ATP per min.

### 3.5. Optimization of Reaction Conditions for Multi-Enzyme Biosynthesis of 3′-SL

The synthesis of 3′-SL from SA and lactose by multi-enzyme biosynthesis was conducted in a 25 mL reaction system with cell-free extracts, substrates, and metal ions. The optimal addition of each cell-free extracts was investigated by varying the wet weight cells from 8–48 g/L. All the other components of the reaction system below were fixed at 50 mM SA, 60 mM lactose, 20 mM polyphosphate, 20 mM CMP, and 20 mM MgCl_2_. The reaction was conducted at 35 °C and pH 7 for 2 h. The samples were collected to detect the 3′-SL content.

Wild-type *E. coli* BL21 Star (DE3) and *E. coli* BL21 Star (DE3) Δ*lac*Z were incubated and processed separately to evaluate the degradation of lactose. The reaction condition included a 40 g/L wet weight of cells which was sonicated on ice bath and 60 mM lactose. The samples were collected at different time intervals to detect the remaining lactose.

The conversion in different pH buffers in the range of 5.0–10.0 was compared, including 50 mM of sodium acetate buffer (pH 5.0–6.0), 50 mM of Tris-HCl buffer (pH 7.0–8.0), and 50 mM of glycine-NaOH (pH 9.0–10.0). The progress of the reaction under different temperatures ranging from 25 °C to 45 °C was also evaluated.

To evaluate the effect of metal ions on the biosynthesis of 3′-SL, 20 mM of storage solution of each metal ion was prepared, including MgCl_2_, CuSO_4_, CoCl_2_, MnSO_4_, ZnSO_4,_ and CaCl_2_. A final concentration of 5 mM of the respective metal ion was used.

Various concentrations of CMP and polyphosphate were investigated to inspect their effect on the production of 3′-SL. Both the substrates were tested at 0 mM, 5 mM, 10 mM, 20 mM, 40 mM, and 60 mM, respectively.

The ratios of four cell extracts were also conducted. To evaluate the ratio between CSS and ST, the addition concentration of CMK and PPK was fixed as 40 g/L (wet weight). The ratios of CSS and ST varying from 5:1 to 1:5, each starting concentration was 40 g/L (wet weight). To investigate the ratios of CMK and PPK, the ratio of CSS and ST was fixed as optimized. The ratios of CMK and PPK range the same as the CSS and ST. All the experiments above were performed in triplicates.

### 3.6. Production of 3′-SL

Briefly, 3′-SL was synthesized in a 100 mL shaker flask with a 25 mL reaction mixture under the optimized conditions. The reaction was incubated at 35 °C in a water-bath with a magnetic stirrer. The reaction mixture contained 50 mM SA, 60 mM lactose, 20 mM MgCl_2_, 20 mM polyphosphate, 20 mM CMP, and 120 g/L CSS and PPK cell-extracts, 40 g/L ST and CMK cell-extracts, and the pH was maintained at 7.0 using 4 N NaOH. The concentration of 3′-SL was detected every 2 h. The reaction was stopped by boiling the system for 2 min, followed by centrifugation at 12,000 rpm for 2 min. The supernatant was diluted to a suitable concentration and detected by HPLC. The experiment was performed in triplicates.

### 3.7. Analytical Methods

Cell density was determined by measuring the optical density at 600 nm with a spectrophotometer (UV-1800, Shimadzu, Suzhou, China). The quantitative analysis of CDP and ATP was performed using HPLC (LC-16, Shimadzu, Kyoto, Japan), which was equipped with a UV detector at 271 nm and a Zorbax C18 column. The mobile phase was 0.6% phosphate-triethylamine (pH 6.6) and the methanol ratio was 89:11. The quantitative analysis of CMP-NeuAc was detected at 210 nm, the mobile phase was 20 mM pH 8.0 phosphate buffer. The samples were detected at 30 °C at a flow rate of 0.6 mL/min.

The concentrations of 3′-SL and SA were measured by HPLC (LC-16, Shimadzu, Kyoto, Japan) which was equipped with a UV detector at 210 nm and a TSK-Gel Amide-80 column. The mobile phase was 10 mM ammonium formate (pH 4.0) and acetonitrile at a ratio of 30:70. Samples were detected at 60 °C at a flow rate of 1.0 mL/min.

The concentration of lactose was measured by HPLC, which was equipped with a refractive index detector and an Aminex HPX-87H column. The mobile phase was 5 mM H_2_SO_4_. The samples were detected at 60 °C at a flow rate of 0.5 mL/min.

## 4. Conclusions

A multi-enzyme cascade for the biosynthesis of 3′-SL and regeneration of CTP was established. It consists of four enzymes expressed in genetically modified *E. coli* BL21 Star (DE3). By increasing the protein solubility and optimizing the reaction conditions, i.e., 35 °C, pH 7, and 20 mM MgCl_2,_ a 97.1% yield of 3′-SL was obtained. CTP was regenerated from low-cost substrates, such as polyphosphate and CMP. The maximum production of 3′-SL was found to be 38.7 mM at 6 h of reaction. The results obtained in this study are meaningful for a cost-effective synthesis of 3′-SL. Coupling multi-enzyme reactions in one pot provide efficient and economical synthesis strategies for the cascade enzyme synthesis of 3′-SL.

## Figures and Tables

**Table 1:** Strains, genes, and plasmids used in the study.

	Description	Reference or Source
**Strains**		
*E. coli* BL21Star (DE3)	F- ompT hsdSB (rB-, mB-) gal dcm rne131 (DE3)	ThermoFisher Scientific
*E. coli* BL21 Star (DE3) △*lac*Z	F- ompT hsdSB (rB-, mB-) gal dcm rne131 (DE3) △*lac*Z	This study
**Genes**		
*css*	CMP-sialic acid synthetase from *Neisseria meningitides* (U60146.1)	[34]
*st*	α-2, 3-sialyltransferase from *Neisseria gonorrhoeae* (U60664.1)	[35]
*cmk*	CMP kinase from *Escherichia coli* (X00785.1)	[36]
*ppk*	Polyphosphate kinase from *Escherichia coli* (CP043942.1)	[37]
**Plasmids**		
pET-CSS	pET-22b (+) containing CMP-sialic acid synthetase	This study
pET-ST	pET-22b (+) containing sialyltransferase	This study
pET-CMK	pET-22b (+) containing CMP kinase	This study
pET-PPK	pET-22b (+) containing polyphosphate kinase	This study

## Supplementary Materials

The following are available online: Figure S1: Optimal addition of cell extracts, Table S1: The relevant results of 3’-SL production, Table S2: Enzyme activities in the cell-free extracts of recombinant strains, Table S3: Description of molecular chaperone expression plasmid.
