# Supplementary material for: Multi-Enzymatic Cascade One-Pot Biosynthesis of 3′-Sialyllactose Using Engineered Escherichia coli
Source: Molecules. 2020 Aug 6;25(16):3567. doi: 10.3390/molecules25163567 (PMC7463868; doi:10.3390/molecules25163567)
Supplement: Supplementary file 1 [file molecules-25-03567-s001.pdf]

Supplementary materials

Table S1 The relevant results of 3′-SL production

Substrate	Enzyme/cell	Time(h)	Content(mM)	Reference
cGMP, lactose	sialidase	1.67	~3.5	10
cGMP, lactose	Trans-sialidase	22	2.4	11
cGMP, lactose	sialyltransferase	6	2.75	12
Orotic acid, lactose, SA	C. ammoniagenes DN510 cells, MM294/pMW6 cells, NM522/pYP3 cells, NM522/pTA23 cells	11	52	13
Lactose, SA, PEP, ATP, CMP	CMP-NeuAc synthetase/sialyltransferase fusion	144	48	14
Pyruvate, ManNAc, CMP, CTP,lactose, polyphosphate	Aggregated enzymes and whole *E.coli* cells.	10	~20	15

Table S2 Enzyme activities in the cell-free extracts of recombinant strains

Strains	Specific activities(U/g)	Background activities(U/g)
E.coli BL21 Star(DE3)Δ*lac*Z/pET-CSS	82.7±5.0	ND
E.coli BL21 Star(DE3)Δ*lac*Z/pET-ST	20.3±3.2	ND
E.coli BL21 Star(DE3)Δ*lac*Z/pET-CMK	807.3±9.1	69.3±2.5
E.coli BL21 Star(DE3)Δ*lac*Z/pET-CMK/pGro7	2737.7±8.5	
E.coli BL21 Star(DE3)Δ*lac*Z/pET-PPK	302.7±7.6	18.7±2.1

Background activities refer to the activity tested by an empty *E.coli* BL21 Star (DE3)Δ*lac*Z strain that does not contain any plasmids; ND: not detected

Table S3 Description of molecular chaperone expression plasmid

Plasmids	Chaperone genes	Promotor	Inducer	Resistance
pG-KJE8	*dnaK-dnaJ-grpE*	araB	L-Arabinose	CM
	groES-groEL	Pzt-1	Tetracycline	
pGro7	*groES-groEL*	araB	L-Arabinose	CM
pKJE7	*dnaK-dnaJ-grpE*	araB	L-Arabinose	CM
pG-Tf2	*groES-groEL-tig*	Pzt-1	Tetracycline	CM
pTf16	*tig*	araB	L-Arabinose	CM

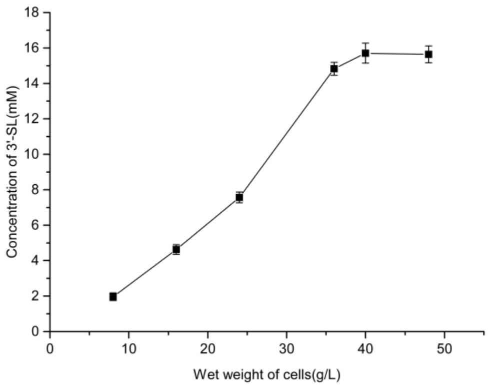

Figure S1 Optimal addition of cell extracts
